# PW02-042 - Induction of MDSC in Muckle-Wells syndrome

**DOI:** 10.1186/1546-0096-11-S1-A183

**Published:** 2013-11-08

**Authors:** N Rieber, A Brand, D Neri, T Hall, I Schäfer, S Hansmann, J Kümmerle-Deschner, D Hartl

**Affiliations:** 1 Children’s Hospital, University of Tübingen, Tübingen, Germany

## Introduction

Muckle-Wells syndrome (MWS) is caused by mutations in the *NLRP3*-gene encoding cryopyrin, leading to overproduction of IL-1β and other NLRP3 inflammasome products. Myeloid-derived suppressor cells (MDSCs) represent a novel innate immune cell subset, are generated in tumor, infective, and proinflammatory microenvironments and are capable of suppressing T cell responses. Consequently, MDSCs are considered a key intermediary in balancing innate and adaptive immune responses, particularly under chronic disease conditions.

## Objectives

We hypothesized that NLRP3 inflammasome-dependent factors induce the generation of MDSCs in MWS.

## Methods

We studied granulocytic MDSC numbers in 25 MWS patients under anti-IL-1 therapy with canakinumab and 20 healthy controls. After Ficoll density gradient sedimentation, granulocytic MDSCs were characterized as CD33^high^CD66b^high^IL-4Ra^inter^HLA-DR^low^ neutrophilic cells in the PBMC fraction, according to previously established human MDSC analysis methods. The functionality of MACS-isolated MDSCs was assessed using polyclonal T cell proliferation and cytokine / chemokine secretion tests. Physician’s global assessment of disease activity, CRP, ESR, and T helper cell subsets were determined at the same time points and correlated with MDSC levels. Serum samples of 22 MWS patients and 5 healthy controls were examined by multiplex technique for possible MDSC inducing factors.

## Results

MWS patients under anti-IL-1 therapy displayed significantly elevated MDSC numbers (mean 1.65 ± 0.33 %; range 0.16 – 5.17 %) compared to healthy controls (mean 0.45 ± 0.05 %; range 0.12 – 1.04%; p = 0.0025), although clinical MWS-disease activity was generally low at time of examination. MDSCs were functionally competent, as they suppressed polyclonal T cell proliferation, Th1, Th2, and Th17 responses. MDSCs correlated directly with Treg/Th17 and Treg/Th1 ratios indicating an influence on T helper cell subsets. Multiplex assays revealed the established MDSC-inducing growth factors GM-CSF and VEGF elevated in MWS sera even under anti-IL-1 therapy with canakinumab.

## Conclusion

MWS patients under anti-IL-1 therapy display significantly elevated numbers of granulocytic MDSCs. Increased MDSCs in MWS might represent a novel autologous anti-inflammatory mechanism in autoinflammatory conditions and may serve as a future therapeutic target.

## Competing interests

N. Rieber Grant / Research Support from: I obtained research grant from Novartis GmbH in 2012, Paid Instructor at: I held a paid talk for Novartis GmbH in 2012, A. Brand: None declared, D. Neri: None declared, T. Hall: None declared, I. Schäfer: None declared, S. Hansmann: None declared, J. Kümmerle-Deschner Grant / Research Support from: Obtained research grants from Novartis GmbH, D. Hartl Grant / Research Support from: Obtained research grants from Novartis GmbH

